# Faeces, Feathers and Flight: Understanding of Escape Behaviour in Incubating Eurasian Woodcocks (*Scolopax rusticola*)

**DOI:** 10.1002/ece3.71573

**Published:** 2025-07-21

**Authors:** Martin Sládeček, Kateřina Brynychová, Lucie Nutilová, Miroslav E. Šálek

**Affiliations:** ^1^ Faculty of Environmental Sciences Czech University of Life Sciences Prague Prague Czech Republic; ^2^ Forestry and Game Management Research Institute Jíloviště Czech Republic

## Abstract

Predators are a leading cause of breeding failure as well as adult mortality in most bird species, prompting the evolution of various antipredator behaviours. Among these, cryptic breeding birds often rely on strategies such as nest concealment and reduced activity to avoid detection. However, even some cryptic species have been observed to respond more actively to an approaching predator. One behaviour suggested to have an antipredatory function is defecating over the nest and eggs when departing. In this study, we investigate this behaviour in incubating female Eurasian woodcocks ( *Scolopax rusticola* ) using a large data set of 399 photographed nests sourced from various open‐source internet platforms. Our analysis reveals that signs of defecation are obvious in 54% of nest photos. Moreover, in 67% of nests are visible freshly moulted feathers are visible around the nest, indicating possible application of fright moulting—a behaviour not previously documented in the context of the antipredator response during escape. We give both these behaviours a common context and suggest that they may help to aid the female's escape rather than to protect the nest, although their antipredatory nature, let alone their effectiveness, remains unclear. The presence of faeces and feathers may, in fact, increase the risk of nest predation by making the nest more conspicuous to predators. Our findings thus also emphasise the importance of minimising nest disturbance during research to preserve the integrity of the nest environment. We show that open‐source platforms can provide valuable data for studies of breeding behaviour in wild birds.

## Graphical abstract

Using a large dataset of nest photographs from open‐source platforms, we investigated defecation and fright moulting in incubating Eurasian woodcocks (*Scolopax rusticola*), finding defecation in 54% of nests and feathers in 67%. We suggest these behaviours may aid the female's escape rather than protect the nest, and highlight the importance of minimising nest disturbance during research.

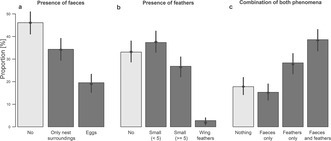

## 1 Introduction

Given that predation is the leading cause of breeding failure for most bird species (Ricklefs 1969), various species have evolved a range of adaptations and mechanisms to mitigate the risk of predation (Caro 2005). These adaptations can be broadly categorised into two strategies. On one hand, they include active nest defence (Larsen et al. 1996), which may be enhanced by forming colonies (Götmark and Andersson 1984; Šálek and Šmilauer 2002). On the other hand, so‐called ‘cryptic breeders’ rely on nest concealment (Sládeček et al. 2015), minimised movement around the nest (Martin et al. 2000) and finely tuned crypsis of both eggs (Colwell et al. 2011; Šálek and Cepáková 2006) and parents (Ekanayake et al. 2015). While some cryptic breeders may actively defend their nests from predators (Halupka and Klimczuk‐Bereziuk 2023; Mérő and Žuljević 2017), most rely on early departure (Sanchez‐Gomez et al. 2024) or freezing until the last moment to avoid detection (Forbes et al. 1994).

One behaviour that has been proposed to have an antipredator function is defecation at the moment of perceived predator approach. This behaviour is commonly observed and well documented in colonial birds that actively defend their nests, such as fieldfares ( *Turdus pilaris* ) (Haas 1985) and various terns and gulls (Fuchs 1977), which attempt to hit approaching predators with falling faeces or vomit (Swennen 1974). In colonial breeders, this behaviour can indeed lead to a reduction in nest predation rates (Andersson and Wiklund 1978; Haas 1985). In extreme cases, it has even been observed that a predator lost the ability to fly due to the intensity of faecal contamination (Bezzel 1975). However, defecation has also been documented in several cryptic breeders, who frequently smear their eggs with faeces when flushed from the nest. Defecation by flushed incubating females is a regular and common occurrence in several duck species, most often in eiders ( *Somateria mollissima* ) (King and Shutler 2010; Swennen 1968) and has also been anecdotally reported in two shorebird species, namely common snipe ( *Gallinago gallinago* ) and great snipe ( *Gallinago media* ) (Müller and Königstedt 1990). Nonetheless, although suggested by several authors (McDougall and Milne 1968; Müller and Königstedt 1990), the antipredatory function and effectiveness of this behaviour remain unclear.

One possible explanation for cryptic breeders defecating in the nest during escape is that the faeces may deter predators by making the eggs unpalatable (McDougall and Milne 1968; Swennen 1968). However, testing the effectiveness of smearing eggs with faeces to deter predators gives contradictory results. While some studies have found a protective effect of faeces against certain predator species (McDougall and Milne 1968; Swennen 1968), other studies reported higher predation rates at faeces‐contaminated nests, likely because predators can use the faeces as an olfactory cue to locate the nest (Clark and Wobeser 1997; Olson and Rohwer 1998). This means that by defecating, the flushed female breaks the olfactory crypsis of the nest. The importance of olfactory crypsis is perhaps best documented by the fact that the incubating sex of many bird species changes the composition of their preen waxes during incubation, replacing more volatile monoesters with less volatile but more costly diesters (Reneerkens et al. 2005, 2007). In addition, we can speculate that faeces spread over the clutch and nest surroundings may also reduce visual crypsis of the nest, as bird predators have been shown to use UV‐reflective spots in urine as visual cues for prey detection (Viitala et al. 1995). Finally, although it may not be essential if it would protect the nest from predation, it should be mentioned that contamination of eggshells with faeces can introduce pathogenic bacteria, posing a risk to the embryo (Cox et al. 2000).

Due to these controversies, an alternative explanation for the adaptive function of defecation has been proposed, suggesting that defecation may protect the escaping female rather than the clutch (King and Shutler 2010). This could be supported by the fact that defecation during flushing is common in various bird species outside the context of incubation (Tillmann 2009). The first hypothesis of how defecation may help the bird escape was that it might lighten the escaping female, which may cause acceleration of her starting speed (Simmons 1955). However, as discussed by King and Shutler (2010), the impact of defecation on the starting speed and maneuverability of duck‐size birds is likely negligible, given the minimal weight of the faeces compared to the weight of the escaping female.

Second hypothesis for how defecation may help the bird escape suggests that falling faeces may serve to divert a predator's attention away from the flushed bird (King and Shutler 2010; Tillmann 2009). A similar idea in a different context was presented by Lindstrom and Nilsson (1988), who described several observations in which a bird escaping from a predator shed a small ‘cloud’ of body feathers even though the predator had not yet touched it. Such behaviour is referred to as ‘fright moulting’ (Lindstrom and Nilsson 1988) but is usually described as autotomic feather loss when the bird is captured by a predator (or handled by humans), similar to the autotomy of the lizard's tail (Awasthy 2010; Dathe 1955). Observation of the feather cloud shedding without direct contact with a predator; however, the authors liken it more to an octopus releasing a cloud of ink (Lindstrom and Nilsson 1988). Within this context, it is noteworthy that dropping faeces and shedding feathers could work in very similar ways, possibly also when an incubating bird is flushed from the nest.

These observations align well with our own experiences, and when encountering Eurasian woodcock ( *Scolopax rusticola* ) nests, as in four out of seven cases, the departing female defecated on or near the eggs while shedding varying amounts of feathers in flight. In one case, only feathers were shed. Eurasian woodcock is a cryptic shorebird inhabiting diverse, primarily forested habitats across its vast breeding range (Hoodless and Hirons 2007; Sládeček et al. 2023). The clutch is incubated exclusively by the female, who remains at the nest for most of the day and night (Trejbalová et al. 2023). Due to her exceptional camouflage and tendency to flush from the nest only when an intruder is within 1–2 m, nests are typically discovered only when the female is startled by an approaching visitor. Finding a nest without the female present, such as during her feeding trips or after predation, is nearly impossible (Hoodless and Coulson 1998). As most nests are discovered accidentally when the female is flushed, any accompanying photographic documentation presented elsewhere is likely taken immediately after her departure. This provides an opportunity to assess the presence of fresh faeces and feathers at the nest site using the photographic material available from various open internet sources, a promising new source of data in biological research (Dylewski et al. 2017; Mikula and Tryjanowski 2016).

Based on the assumed context and circumstances surrounding the usual photographs of woodcock nests, we analysed photographs of woodcock nests taken across the species' breeding range in Eurasia, using freely available online sources. We examined the presence of easily detectable faeces and feathers within the nests and their immediate surroundings. Specifically, we assessed their frequency of occurrence and tested the likelihood of their co‐occurrence. Based on previous findings, we assumed that most photographs were taken immediately after the female was flushed from the nest and that the well‐visible faeces and/or feathers present were deposited at the moment of female departure.

We suggest that if fresh faeces frequently appear in the images, their deposition is related to an antipredator response triggered by the female's escape from the nest, as previously documented in some ducks and episodically in two shorebird species related to woodcocks (see above). Moreover, if feathers are also shed at the same time, we infer this may represent a phenomenon called ‘fright moulting’, which has not yet been described in relation with the escape behaviour of incubating birds (Lindstrom and Nilsson 1988). Therefore, if the presence of faeces and feathers at the nests both accompany the female's forced departure, that is, if they serve a common function, we hypothesise that their presence in nest photographs should be correlated. We discuss our findings and their possible interpretations in detail.

## 2 Methods

### 2.1 Data Searching and Filtering

To assess the behaviour of flushed breeding woodcocks, we combined several sources of photos. First, we conducted an extensive search of the various open sources on the internet, including literature searches, searches of faunistic databases operating in countries throughout the breeding range of the Eurasian woodcock, and also searches of Google images, a number of social media and networks (see Table A1 for the list of all sources and the number of nests obtained). Second, we supplemented this data set with nests that we found in the past, our colleagues, or friends who provided us with photographs of nests they found (*n* = 7). Finally, we included the only picture that we obtained based on a public call published in the hunting magazine ‘Svět myslivosti’ (*n* = 1).

In all faunistic databases (see Table A2 for the list of databases and number of nests obtained), we filtered and manually checked all photos of Eurasian woodcock nests with eggs or incubating females taken in any year between 1 March and 31 August, which (with a certain margin) covers the presumed breeding season of the woodcock. Photographs of nests published in the literature were searched through the Google scholar database (see Table A3 for the list of literature sources used).

We searched Google images and social media/networks in most of the national languages used throughout the breeding range of the Eurasian woodcock (see Table A4 for the list of languages), and the scientific name *Scolopax rusticola* . In all languages, we used queries that can be translated into English as ‘woodcock's nest’ and ‘woodcock eggs’ (see Table A4 for the list the queries used). If we found more than one photograph for a single nest, we included all of them. Within YouTube, we extracted photographs from each video by cropping them with the ‘print screen’ function. In this case, we tried to cut out photographs showing both the whole (surrounding habitat) and a detail of the nest.

Once we found a photograph of the nest, we tried to find as much traceable information as possible. In particular, the location where the photograph was taken (at the finest possible level of geographical detail), the date when the photograph was taken, the author of the photograph, and possibly other details about the circumstances surrounding the discovery of the nest. For this, we tried to use any information from the website/social media post, or information from other posts/discussions, etc.

Google image searches were stopped at the end of 2023; social media and databases were checked until 30 June 2024. On the other hand, the oldest photograph found suitable for the analysis in this article was taken in 1969. The individual image sources were divided among three authors (MS, KB, LN) who searched them independently. The first author then merged and cleaned all the data sets obtained in this way. Every possible effort was made, especially to ensure the exclusion of duplicate images of the same nest, nests of another species and photographs of artificially arranged museum exhibits. Specifically, before inclusion in the data set, each found photograph was carefully compared to all previously found photographs from the same country and similar time period previously acquired from any other source.

### 2.2 Key Assumptions Supporting the Data Interpretation

Interpretation of observed phenomena is based on several key assumptions that we consider well supported by biological reasoning, field observations and existing literature.

First, we assume that most woodcock nests are found when the incubating female is flushed and are active at the time of discovery. Given our experience and previous studies, almost all nests are found incidentally when the incubating female is flushed. This was the case for every nest we located, for all nests reported by our collaborators, and for those described in sources from which we obtained photographic material, whenever this information was available. Moreover, an analysis of approximately 460 breeding records shows that only 2% were found to be nonactive (predated/abandoned) (Hoodless and Coulson 1998). This suggests that nearly all found woodcock nests were active at the moment of discovery. Thus, we consider it reasonable to assume that the vast majority of photographs used in our study were taken immediately after the female's departure, reflecting the nest's condition at that moment.

Second, we assume that the faeces and feathers found at the nest originate from the incubating female rather than from other birds or predators and have not been dropped during incubation (i.e., in situations unrelated to the flushing female from the nest). Woodcocks breed solitarily, and only the female incubates, making it highly unlikely that other individuals regularly visit the nest. Our field observations support this assumption, as in five out of seven cases, we directly observed defecation and/or feather shedding when the female was flushed. Additionally, as woodcocks rely heavily on their fine‐tuned crypsis, we consider it highly unlikely that they would defecate in their nests or in close surroundings to them without a strong stimulus, such as forced flight when flushed, thereby voluntarily increasing the visual conspicuity of the nest's immediate surroundings.

Finally, we assume that the presence of faeces or feathers does not make nests more detectable to human vision. As the vast majority of nests in our data set were discovered incidentally when the incubating female was flushed, it is unlikely that small droppings or scattered feathers significantly influenced their detection. Moreover, many nests were found by human visitors without a particular focus on birds, further reducing the likelihood that such subtle signs contributed to nest discovery. Therefore, there is no reason to believe that nests with faeces or feathers were overrepresented in our data set due to increased visibility.

### 2.3 Photo Evaluation and Data Analysis

All the work with the data, including picture inspection, data curation, statistics calculation and visualisation, was carried out in R version 4.2.1 (R‐Core‐Team 2022). One of the authors (MS) carefully examined all obtained photographs, recording the presence of faeces and feathers. Both variables were recorded as NA if the image quality was too poor to reliably assess both observed characteristics (e.g., the nest was photographed from too far away or had poor resolution). Additionally, since the goal was to evaluate nest conditions after the incubation female had been flushed, photographs showing the female sitting on the nest were excluded from the analysis.

**FIGURE 1:**
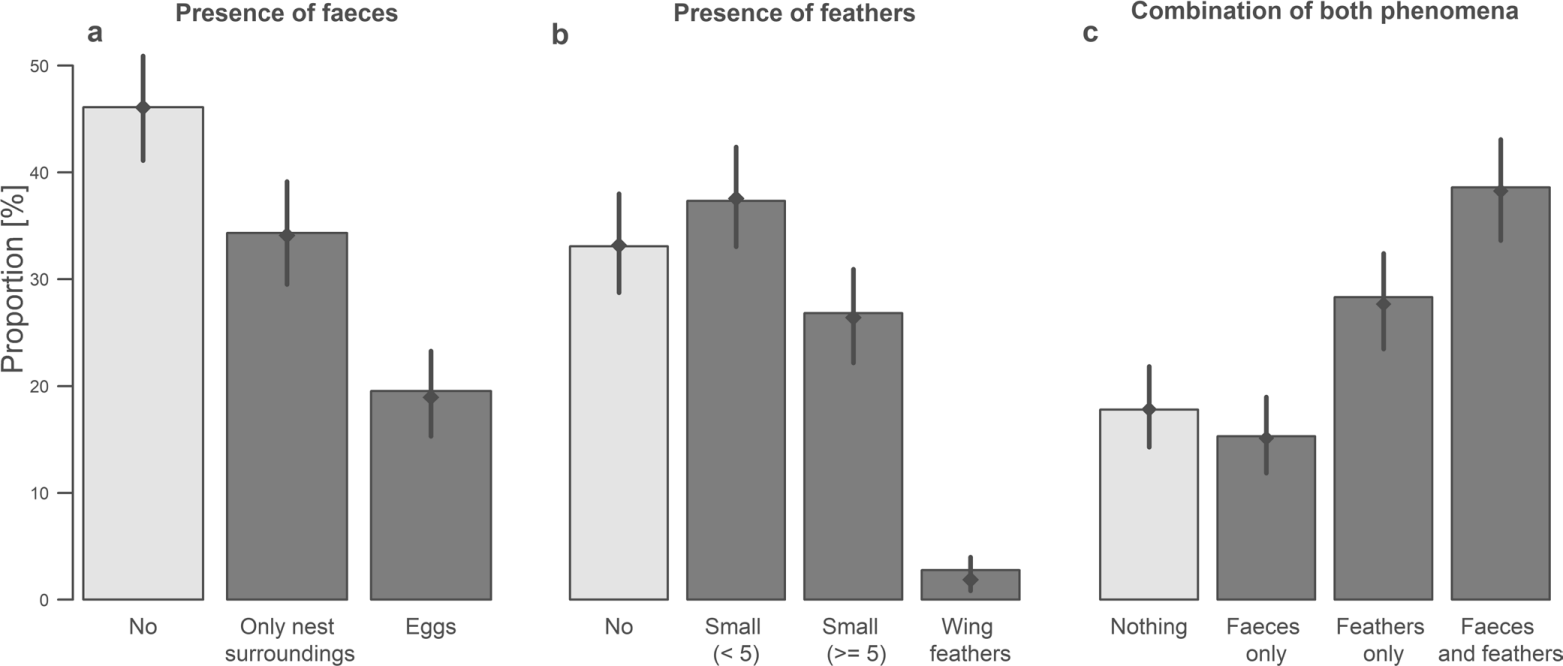
Proportions of faeces occurrence and location (a), feathers, their type and abundance (b), and the representation of each combination of both phenomena found in the entire data set evaluated (c) (*n* = 399 nests). The bars represent the proportions found in the original data set, while the points and error bars represent the estimates based on GLMs and their 95% credible intervals (i.e., after controlling for the basic characteristics of the assessed image).

**FIGURE 2:**
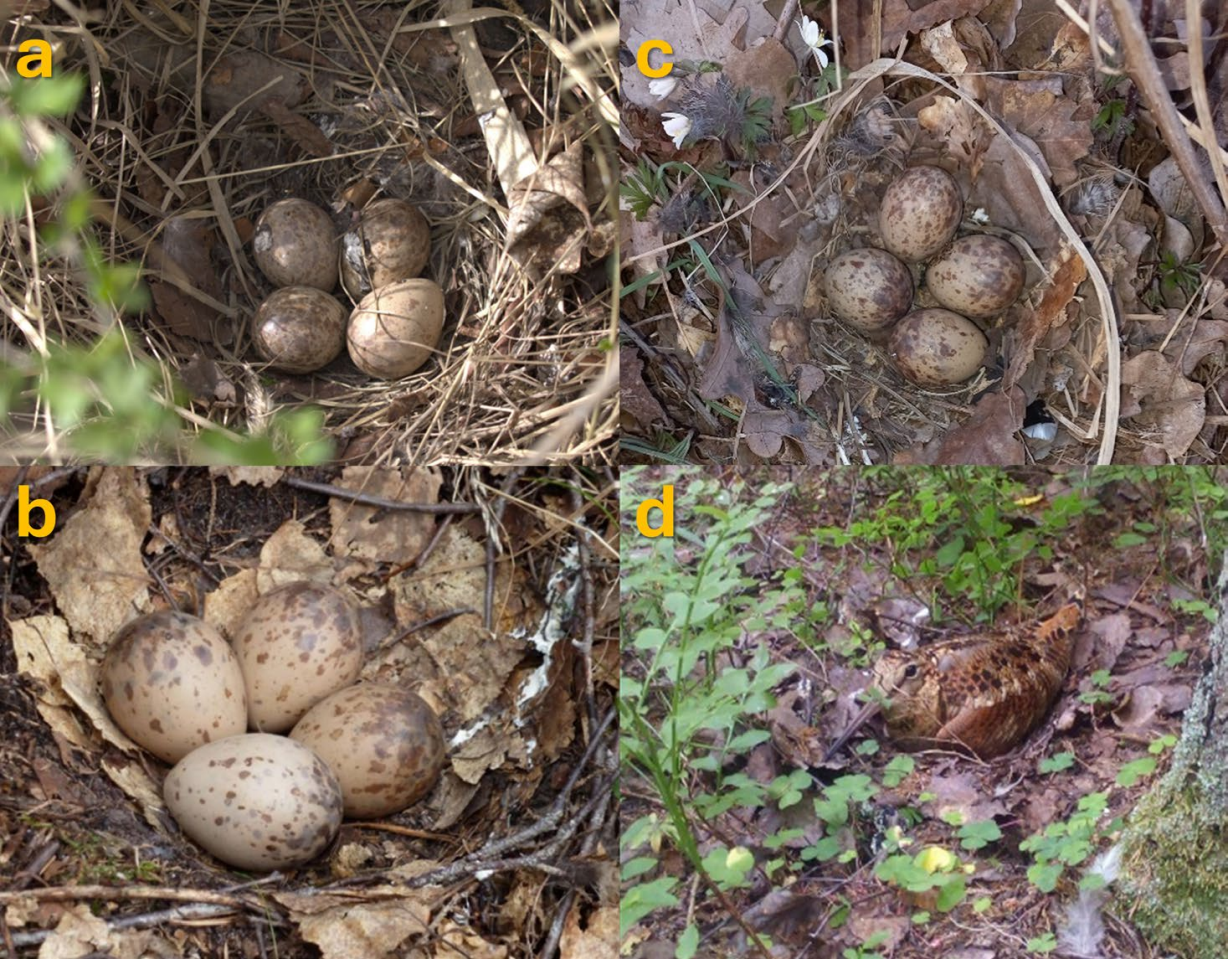
Examples of analysed photos; (a) nest with eggs contaminated with faeces (photo credit: Miroslav E. Šálek), (b) nest with only the surroundings contaminated with faeces (Source: EBird; photo credit: Stephen Carter), (c) nest with several small feathers visible (photo credit: Karel Pithart), and (d) female woodcock incubating nest with clearly visible faeces traces (and one feather) around the nest (Source: Instagram; photo credit: Aniuta Gorchakova).

First of all, we distinguished whether there were signs of faeces in the photograph. Specifically, we distinguish three categories of faecal contamination (Figure 1a). The first category was direct faecal contamination of eggs. The second category was assigned when eggs were not directly affected, but there were faeces in the nest surroundings visible in the photograph (Figure 2). In such cases, contamination usually begins several centimetres from the eggs, although sometimes it could begin at a distance of up to a few tens of centimetres. See, however, below that including the frame size in the analyses does not lead to significant changes in the estimated proportion of nests with faecal contamination of nest surroundings. The third category was assigned when both the nest and its visible surroundings were without marks of recent faecal contamination, but there were visible small white dry specks of faeces on the eggs. However, the interpretation of this category is unclear. It may be the result of faecal contamination during a female's interruption in the past as well as faecal contamination during egg laying. Therefore, we did not use this category in the following analyses.

The presence of feathers was also recorded in three categories. The first category included cases where only a few (< 5) small body feathers (down or contour feathers) were present. The second category was assigned when five or more small body feathers were clearly identifiable in the photograph, as we assume such a situation is highly unlikely without immediate feather shedding during escape. The third category included cases where at least one flight feather was also present (Figure 2). The flight feathers were all from the wing, specifically primary or secondary feathers. Notably, flight feathers were always accompanied by five or more small body feathers, meaning the third category represents a higher level than the second.

The photographs used in this study varied significantly in terms of distance, angle, frame width and image quality, all of which could influence the detectability of recorded phenomena. Additionally, some images may have been altered by photographers, for example, by removing faeces from eggs to enhance aesthetic appeal. Such factors could impact the accuracy of the data, particularly as the number of feathers visible in a photograph is likely lower than the actual number present in the nest surroundings. These limitations are acknowledged and discussed further in the discussion section.

To systematically document image characteristics and control for these variations, we employed a semi‐automated script. Using the ‘locator’ function, we recorded the coordinates of four edge points of the nest scrape (leftmost, lowest, rightmost and highest) in each image. These coordinates were then used to calculate key image characteristics, ensuring a standardised approach to assessing detectability:

1. Nest width (in pixels): determined as the x‐coordinate difference between the rightmost and leftmost points. Since all nests were assumed to be of similar size, this served as a proxy for image resolution.
2. Nest height (in pixels): calculated as the y‐coordinate difference between the highest and lowest points.
3. Frame angle: derived using the formula 90‐ arccos (nest height/nest width) × (180/π), where 90° indicates a top‐down (perpendicular) view, and 0° represents a side view.
4. Relative frame width: computed as the ratio of total image width to nest width.
5. Relative frame height: computed as the ratio of total image height to nest height.

Then, relative frame width, relative frame height, nest width (as proxy for image resolution), and frame angle were used to standardise all estimated proportions of faeces/feathers occurrence, either generally or for particular categories. For each proportion, we fitted binomial models using the *glm* function with logit link function (R‐Core‐Team 2022). The response variable was binary: 1 if a particular event was detected and 0 if not. Predictors included relative frame width, relative frame height, image resolution and frame angle. All predictors were z‐standardised (mean centred and divided by SD), which, in addition to better comparability of their effect sizes (Schielzeth 2010), allowed obtaining estimates of individual proportions for ‘standardised photo’ Specifically, the intercept of the model is estimated for an ‘average photo’—that is, a shot that has the average values of all the parameters that the model controls for. Note, however, that none of the explanatory variables had a significant impact on detection probability for the majority of estimated proportions (see Table A5). For all the commented proportions, we then report the estimate and its 95% Bayesian credible intervals obtained from a posterior distribution of 5000 simulated values generated by the ‘sim’ function from library ‘arm’ (Gelman et al. 2016).

To test the independence of occurrence between both observed phenomena, we used the chi‐squared test of independence with Yates continuity correction.

## 3 Results

**MAP A1:**
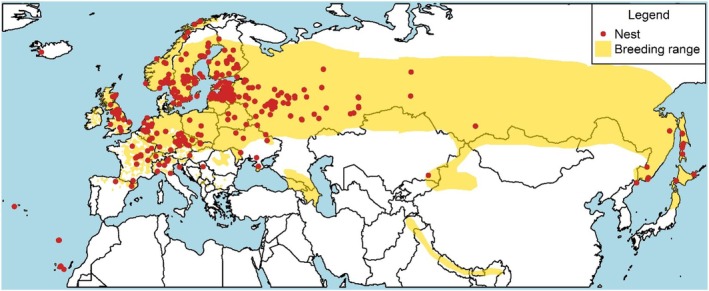
Map showing the European woodcock breeding range and positions of all nests included in the analysis (*n* = 399). BirdLife International data and Handbook of the Birds of the World (2023) Maps of the distribution of bird species in the world were used as a basis for the breeding range. Version 2023.1. Available at http://datazone.birdlife.org/species/requestdis.

A total of 658 photographs of 501 nests were obtained. Of these, for 399 nests, we obtained at least one image that allowed us to assess whether the nest or its immediate surroundings were contaminated with faeces and whether there were any feathers (Map A1).

In total, fresh faeces indicating female defecation shortly before the picture was taken were observed in 215 nests (glm estimate: 60%; 95% CrI: 49%–59%, Table A5a). Of this number, however, only in 78 cases (glm estimate 35.7%; 95% CrI: 29%–43%; Figure 1a, Table A5b) was at least one of the eggs contaminated. In the remaining cases, only the immediate surroundings of the nest were contaminated. The amount of visible faeces, as well as the extent to which the eggs or surroundings were affected, was highly variable.

In 267 nests (glm estimate 67%; 95% CrI: 62%–71%, Table A5c) or their surroundings, we observed at least one feather. In the vast majority of such cases (256; glm estimate 97%; 95% CrI: 94%–99%; Figure 1b, Table A5d), these were small body feathers, and in 118 cases (glm estimate 40%; 95% CrI: 34%–46%; Figure 1b, Table A5e), there were five or more visible small body feathers within the photo. In addition to five or more small body feathers, at least one large flight feather (primaries or secondaries) was observed on the nest in 11 cases (glm estimate 3%; 95% CrI: 1%–6%; Figure 1b, Table A5f).

Taken together, only in 71 nests (glm estimate 18%; 95% CrI: 14%–22%; Figure 1c, Table A5g) had neither faeces nor feathers observed. On the contrary, in 154 nests both faeces and feathers were found (glm estimate 38%; 95% CrI: 34%–43%; Figure 1c, Table A5h). Faeces and feathers showed a slightly significant co‐occurrence pattern, with nests being more likely to contain both (or neither) compared to cases where only one was present (Chi = 4.22, df = 1, *p* value =0.039).

## 4 Discussion

In this study, we analysed extensive photographic material from various open‐source internet sources to examine traces of faeces and feathers in and around nests, aiming to demonstrate that defecation and fright moulting often accompany the escape behaviour of female Eurasian woodcocks flushed from their nests. Specifically, we observed signs of defecation in 54% of nests and feathers around the nest visible in 67% of nests, suggesting possible fright moulting during nest departure.

### 4.1 Methodological Limitations

While the occurrence of fright moulting in this context has not been previously inferred, the frequency of defecation can be compared with similar behaviours observed in several duck species, which defecate (and smear the eggs) almost any time they are flushed, as quantified by Swennen (1968). However, it is important to acknowledge the limitations of relying on photographic material from internet sources, even though it remains the most feasible approach for studying these phenomena, given the extraordinary difficulty of locating nests of a highly cryptic and elusive species such as the Eurasian woodcock. Our estimates of the frequency of defecation, as well as the presence and quantity of feathers around the nest, are almost surely underestimated for several reasons. First, faeces and feathers may be dispersed over a larger area than is visible in the photographs, which often focus only on a nest and its immediate surroundings. Second, although it is reasonable to assume that the majority of nests were photographed immediately after the female was flushed, the exact circumstances under which the photographs were taken are not always known, and some images may have been captured in a different context. Third, in some cases, it appears that the nest and eggs may have been ‘cleaned’ by the photographer before the photo was taken, in order to improve its aesthetic quality. This would also reduce the likelihood of detecting both studied phenomena. However, despite these limitations it is important to emphasise that our data set is likely the largest ever compiled for this species. Finding Eurasian woodcock nests is extremely challenging and collecting representative data on their breeding biology through direct fieldwork is nearly impossible, making our findings particularly valuable.

Nonetheless, the lower frequency of defecation observed in woodcocks compared to ducks, even after accounting for the methodological biases described above, may also have a biological explanation. Unlike ducks, which often incubate almost continuously (Bolduc and Guillemette 2003), woodcock interrupt incubation several times during daylight hours (Trejbalová et al. 2023), when most photographs were taken, allowing them to defecate more frequently than ducks. Some females may have defecated shortly before the photograph was taken, reducing the likelihood of defecating again within a short period.

Another issue to address is the uncertainty about the origin of feathers found around the nest. We cannot definitively conclude that they are the result of fright moulting, as some may have come from regular partial moulting, which can overlap with the early breeding season (Hudec and Šťastný 2005). However, feathers were frequently observed throughout the breeding season, and many photographs clearly showed a relatively large number of feathers scattered around the nest shortly before the images were taken. Additionally, partial moulting into breeding plumage should not involve flight feathers, yet these were observed in 4% of nests. Moreover, our extensive experience with several shorebird species that escape from the nest from a safe distance, such as the northern lapwing ( *Vanellus vanellus* ) and little ringed plover ( *Charadrius dubius* ), provides a contrasting perspective. Although these species also undergo regular moulting during part of the breeding season (Hudec and Šťastný 2005), using the extensive photographic material obtained in our previous research activities, we have not been able to find even a single instance of a single feather on or near the nest (own unpublished results, *n* = 159 nests of northern lapwing and 84 nests of little ringed plover).

### 4.2 Possible Functions of Defecation and Fright Moulting

Our study suggests that defecation, often accompanied by the shedding of feathers, is a common behaviour in woodcocks when flushed from the nest.

The simplest explanation for these behaviours is that they are physiological by‐products of an acute stress response triggered by the activation of the sympathetic nervous system, which is necessary to minimise reaction time when the bird is threatened by a predator (Jerem and Romero 2023; Romero and Wingfield 2016). Frequent defecation often accompanies the stress induced by handling (Boissy 1995; Romero and Wingfield 2016), or predator model presentation (Clarke et al. 2012). Easy shedding of feathers during fright moult may be related to the reduction in surface temperature (reviewed in: Jerem and Romero 2023), that occurs as a result of constriction of the visceral vasculature, which is necessary to increase perfusion pressure and redirect blood flow to where it is most needed—the skeletal muscles (Vianna and Carrive 2005). Notably, feather loss is a regular response of male Eurasian woodcock caught in mist‐nets (own observations).

The key question for this hypothesis is whether a female's escape from the nest is perceived as an immediate life‐threatening situation sufficient to trigger the acute stress response (Romero and Wingfield 2016). This was disputed by Tillmann (2009) in his study of flushed coveys of grey partridges ( *Perdix perdix* ). Although we did not test this in female woodcocks, we consider it highly probable, as they typically rely on crypsis and remain on the nest until the intruder is very close, often within a metre. Importantly, this behaviour is very similar to what is observed in other species where defecation upon being flushed from the nest has been documented (Müller and Königstedt 1990; Swennen 1968). The urgency and stress of such an escape are likely significant, especially given the proximity of potential predators capable of directly attacking the sitting female.

The question remains whether this behaviour has an adaptive function in reducing nest predation (by making the eggs unpalatable; Swennen 1968) or female predation (by distracting an attacking predator; King and Shutler 2010; Tillmann 2009). While we lack direct data on the risk of female predation during incubation, nest predation rates in woodcocks are reportedly high (Hoodless and Coulson 1998). However, even if this behaviour did not evolve primarily as a response to predator threats but rather as a physiological byproduct of stress, any potential impact on an attacking predator could still be a beneficial side effect, even if its effectiveness is low.

Nevertheless, we propose that frequent defecation by female Eurasian woodcocks is unlikely to function as an effective adaptation for nest protection for several reasons. First, a significant proportion of dropped faeces is deposited next to the nest, meaning the eggs themselves are not directly affected and are unlikely to be protected. Second, in ducks, the protective effect of faeces, if any, is specifically linked to the type of faeces produced by birds during incubation. The predator deterrent effect disappeared when the faeces of nonbreeding birds were used in experiments (Swennen 1968). This is a consequence of the fact that almost continuously incubating ducks fast during the incubation (Bolduc and Guillemette 2003). This causes them to produce very specific faeces with a modified bacterial composition and more alkaline pH (McDougall and Milne 1968), which are likely unpalatable to many predators (Swennen 1968). Although we did not measure the specific characteristics of the faeces produced by incubating Eurasian woodcock, we assume that it will not differ from the faeces of nonincubating birds, as the incubation rhythm of Eurasian woodcock allows females to avoid fasting (Trejbalová et al. 2023). The protective potential of their faeces for eggs is thus rather improbable.

Third, the presence of faeces and conspicuous urine spots around the nest likely makes the nest more noticeable to a wide range of potential predators. This increased noticeability could result from reduced olfactory crypsis (Reneerkens et al. 2005), attracting mammalian predators (Clark and Wobeser 1997; Olson and Rohwer 1998), as well as using UV‐reflective urine spots as visual cues by avian predators to locate prey (Viitala et al. 1995). The latter is particularly relevant during incubation breaks, when the nest remains uncovered for about 3 h each day during daylight (Trejbalová et al. 2023). Notably, feathers scattered outside the nest could pose a similar problem in terms of crypsis.

Fourth, woodcocks regularly abandon the nest completely after being flushed, especially during the first third of the incubation period. The probability of nest desertion after being flushed is about 12% (Hoodless and Coulson 1998). It is thus possible that the primary focus of the escaping female may be on preserving her own life, rather than protecting the eggs.

Given the above reasoning, we consider it likely that if defecation by a woodcock female flushed from the nest has a specific adaptive function, it helps her safely escape predation, most likely by diverting the predator's attention (King and Shutler 2010). This hypothesis places defecation and fright moulting, the two phenomena inferred in this paper, in a common context: in order to distract the predator, the flushed female may leave behind either faeces, a small cloud of feathers or even both. This is also consistent with the fact that defecation when flushed has also been observed in many birds outside the incubation context (Reviewed in: Tillmann 2009).

### 4.3 Conclusions and Future Directions

Our study suggests new perspectives on the behaviour of cryptic breeding birds when flushed from their nests. In particular, we highlight the occurrence of possible fright moulting, a phenomenon not previously documented in this context, in addition to the already known defecation. Our results suggest several directions for future research. To begin with, while our findings focus on Eurasian woodcock, and defecation has been observed in several other species, the prevalence and distribution of these two phenomena across avian taxa remain largely unexplored. We hypothesise that both phenomena will be most (or exclusively) present in species that rely on crypsis and escape only when a predator (or human) approaches from the immediate vicinity. In addition, the physiological mechanisms underlying these behaviours, including their potential link to sympathetic nervous system activation, require further investigation.

Generally, we propose that avian nesting biology could be another field where open‐source internet platforms provide valuable opportunities for data mining in future studies, both for single‐species analyses and comparative studies across multiple species.

Finally, our findings may also have conservation implications. Since the dispersal of faeces or feathers around the nest is unlikely to provide egg protection and may instead increase the risk of subsequent predation, we recommend minimising the number of nest visits during research, especially in species that exhibit defecation and feather shedding when flushed from the nest. If possible, nest visits should be scheduled to coincide with periods when the female is away from the nest during an incubation break. Given that disturbances to woodcock nests are often accidental and unintentional, it may be worth considering at least basic cleaning of the nest and its surroundings if contaminated with faeces and/or a large number of feathers after a visit.

## Author Contributions

**Martin Sládeček:** conceptualization (lead), data curation (lead), formal analysis (lead), funding acquisition (lead), investigation (lead), methodology (lead), project administration (lead), resources (lead), software (lead), visualization (lead), writing – original draft (lead), writing – review and editing (equal). **Kateřina Brynychová:** data curation (equal), investigation (equal), writing – review and editing (equal). **Lucie Nutilová:** data curation (equal), investigation (equal), writing – review and editing (equal). **Miroslav E. Šálek:** data curation (supporting), supervision (lead), writing – review and editing (equal).

## Conflicts of Interest

The authors declare no conflicts of interest.

## Data Availability

Used data set and computer code to replicate our results are freely available from OSF: https://osf.io/ys8hz/.

## Appendix A

**TABLE A1:** List of sources used to search for nest images (except of faunistic databases) and corresponding number of nests found.

Social media and networks	Number of nest images
Facebook	32
Flickr	22
Google Images	109
Instagram	50
Twitter	30
YouTube	6
**Published literature**	17
**Team**	7

**TABLE A2:** List of faunistic databases used to search for nest images, their geographical coverage and number of nests found.

Database	Link	Coverage	Number of nests
Avif	https://avif.birds.cz/	Czech Republic	7
Artportalen	https://artportalen.se/	Sweden	24
Artsobservasjoner	https://www.artsobservasjoner.no/	Norway	19
BirdPhoto	http://www.birdphoto.cz/	Czech Republic	3
Birding Slovakia	https://birding.sk/	Slovakia	1
Dabasdati	https://dabasdati.lv/	Latvia	41
eBird	https://ebird.org/	Worldwide	5
Elurikkus	https://elurikkus.ee/	Estonia	9
iNaturalist	https://www.inaturalist.org/	Worldwide	71
NDOP	https://portal23.nature.cz/	Czech Republic	3
Ornitho	https://www.ornitho.at/	Austria	2
Ornitho	https://www.ornitho.cat/	Catalonia	1
Ornitho	https://www.ornitho.de/	Germany	12
Ornitho	https://www.ornitho.ch/	Switzerland	7
Ornitho	https://www.ornitho.it/	Italy	3
Ornitho	https://www.ornitho.pl/	Poland	6
Russian Bird Conservation Union	https://rbcu.ru/	Russia	1
Ukrainian Biodiversity Information Network	https://ukrbin.com/	Ukraine	2
waarnemingen	https://waarnemingen.be/	Belgium	4
waarnemingen	https://waarneming.nl/	Netherlands	6

**TABLE A3:** List of literature sources containing photo material used in this publication.

Belik, K., & Beuch, S. (2019). Exceptionally early breeding of Woodcock *Scolopax rusticola* in Lubliniec Forest (Silesian Vivodeship). Ptaki Śląska, 26, 127–131.
Gritschik, V.V., Sandakov, S.D., Mindlin, G.A., Vorobjev, V.N. (2011). Woodcock ( *Scolopax rusticola* ) biology in Belarus. 2. Breeding biology. Subbuteo, 10, 3–10.
Glushchenko, Y.N., Korobov, D.V., Shokhrin, V.P., Vyalkov, A.V., Sotnikov, V.N., Tiunov, I.M., Bachurin, G.N. (2023). Materials to the study of woodcock *Scolopax rusticola* in the south of the Russian Far East. Russian Journal of Ornithology, 32(2278), 819–838.
Grigoriev, E.V. (2016). Finds of nests of woodcock *Scolopax rusticola* in the Novorzhevsky district of the Pskov region. Russian Journal of Ornithology, 25(1356), 4153–4156.
Grigoriev, E.V. (2017). Late clutches of woodcock *Scolopax rusticola* in the Novorzhevsky district of the Pskov region. Russian Journal of Ornithology, 26(1482), 3292–3295.
Golovan, V.I. (2012). Birds of the vicinity of the village Krasnitsy (Gatchina district of the Leningrad region). Russian Ornithological Journal, 21(750), 899–927.
Hoodless, A. (1995). Studies of west palearctic birds. 195. Eurasian Woodcock *Scolopax rusticola* . British Birds, 88(12), 578–592.
Morgan, R., & Shorten, M. (1974). Breeding of the Woodcock in Britain. Bird Study, 21(3), 193–199.
Rubines, J., & Gómez, B. (2006). Técnicas de caracterización genética de poblaciones de becada *Scolopax rusticola*. En, J. M. Fernández (Ed.): Actas del Encuentro de Ornitología en Álava, pp. 94–100. Diputación Foral de Álava. Vitoria.
Steinfatt, O. (1938). Das Brutleben der Waldschnepfe. Journal für Ornithologie, 86(3), 379–424.
Zekhuis, M. J. (2015). De Houtsnip *Scolopax rusticola* en haar geheimen. Het Vogeljaar, 63(3), 140–146.

**TABLE A4:** All search languages and queries that can be translated into English as ‘woodcock's nest’ and ‘woodcock's eggs’.

Language list	Query
Belarusian	Гняздо вальдшнепа
	Яйка вальдшнепа
Bulgarian	Гнездо Горска бекасина
	яйца Горска бекасина
Croatian	Gnijezdo šljuke
	Jaje šljuke
Czech	Hnízdo sluky lesní
	Vejce sluky lesní
Danish	Skovsneppe rede
	Skovsneppe æg
Dutch	Houtsnipnest
	Houtsnipei
English	Woodcock nest
	Woodcock eggs
Estonian	Metskurvitsa pesa
	Metskurvitsa muna
Finnish	Lehtokurpan pesä
	Lehtokurpan muna
French	Nid de bécasse des bois
	Œuf de bécasse des bois
German	Waldschnepfennest
	Waldschnepfeneier
Hungarian	Erdei szalonka fészke
	Erdei szalonka tojása
Italian	Nido di beccaccia
	Uovo di beccaccia
Japanese	ウッドコックの巣
	ウッドコックの卵
Latvian	Sloka Ligzdo
	Sloka olas
Lithuanian	eurazinė slanka lizdas
	eurazinė slanka kiaušinis
Norwegian	Rugdenes rede
	Rugdeegg
Polish	Gniazdo słonki
	Jajo słonki
Portuguese	Ninho de Galinholas
	Ovo de Scolopax rusticola
Romanian	cuib Sitar de pădure
	ouă Sitar de pădure
Russian	Гнездо вальдшнепа
	Яйца вальдшнепа
Serbian	Гнездо шљуке
	Јаје шљуке
Slovenian	Gnezdo lesne šljuke
	Jajce lesne šljuke
Slovakia	hniezdo sluka hôrna
	Vajcia sluka hôrna
Spanish	Nido de becada
	Huevo de becada
Swedish	Morkullans bo
	Morkullans ägg
Ukrainian	гніздо вальдшнепа
	Яйця вальдшнепа

*Note:* The scientific name *Scolopax rusticola* was also included in the search. For use in Facebook and Instagram, the queries were used also in the form of hashtags, for example, #woodcockegg, #woodcocknest.

**TABLE A5:** Posterior estimates (medians) of the effect sizes with the 95% credible intervals (CrIs) from a posterior distribution of 5000 simulated values generated by the ‘sim’ function in R (Gelman et al. 2016).

Effect	Estimate	95% CrI	
		Lower	Upper
**(a) Overall probability of faecal contamination of eggs or nest surroundings**			
Intercept	0.159	−0.044	0.356
Resolution	0.093	−0.139	0.32
Picture angle	−0.039	−0.24	0.167
**Width rel**.	**0.353**	**0.043**	**0.669**
Height rel.	−0.124	−0.416	0.176
**(b) Probability of eggs being hit if nest was contaminated with faeces**			
**Intercept**	**−0.59**	**−0.873**	**−0.307**
Resolution	−0.08	−0.363	0.213
Picture angle	−0.121	−0.419	0.189
Width rel.	0.106	−0.325	0.539
**Height rel**.	**−0.543**	**−1.043**	**−0.035**
**(c) Overall probability of at least 1 feather in nest or nest surroundings**			
**Intercept**	**0.702**	**0.501**	**0.918**
Resolution	0.151	−0.115	0.419
Picture angle	0.104	−0.112	0.326
Width rel.	−0.08	−0.373	0.217
Height rel.	0.014	−0.273	0.304
**(d) Probability that all feathers were small body feathers, given at least one feather was present**.			
**Intercept**	**3.514**	**2.74**	**4.305**
Resolution	0.006	−0.799	0.797
Picture angle	−0.583	−1.26	0.101
Width rel.	0.733	−0.436	1.896
**Height rel**.	**−0.85**	**−1.648**	**−0.081**
**(e) Probability that there were more than 5 feathers, given at least one was present**.			
**Intercept**	**−0.424**	**−0.664**	**−0.169**
Resolution	0.116	−0.147	0.377
Picture angle	0.065	−0.192	0.323
Width rel.	0.008	−0.352	0.367
Height rel.	0.157	−0.227	0.543
**(f) Probability that there were also at least 1 flying feather, given at least one feather was present**.			
**Intercept**	**−3.536**	**−4.306**	**−2.765**
Resolution	−0.001	−0.809	0.787
Picture angle	0.586	−0.067	1.237
Width rel.	−0.734	−1.904	0.424
**Height rel**.	**0.869**	**0.074**	**1.631**
**(g) Probability that neither faeces nor feathers were found**			
**Intercept**	**−1.527**	**−1.783**	**−1.267**
Resolution	−0.137	−0.467	0.2
Picture angle	−0.08	−0.35	0.189
Width rel.	0.04	−0.319	0.418
Height rel.	−0.148	−0.532	0.223
**(h) Probability that both faeces and feathers were found**			
Intercept	−0.478	−0.684	−0.273
Resolution	0.139	−0.098	0.371
Picture angle	0.006	−0.213	0.216
Width rel.	0.29	−0.009	0.584
Height rel.	−0.195	−0.486	0.11

*Note:* Variance components were estimated by the ‘glm’ function for binomial errors with the logit link function. All continuous covariates were z‐transformed (mean‐centred and divided by SD). Estimates whose 95% CrIs did not contain 0 are highlighted in bold.
